# A mobile brain-body imaging dataset recorded during treadmill walking with a brain-computer interface

**DOI:** 10.1038/sdata.2018.74

**Published:** 2018-04-24

**Authors:** Yongtian He, Trieu Phat Luu, Kevin Nathan, Sho Nakagome, Jose L. Contreras-Vidal

**Affiliations:** 1 Noninvasive Brain-Machine Interface System Laboratory, University of Houston, Houston, TX 77004, USA

**Keywords:** Motor control, Brain-machine interface, Regeneration and repair in the nervous system

## Abstract

We present a mobile brain-body imaging (MoBI) dataset acquired during treadmill walking in a brain-computer interface (BCI) task. The data were collected from eight healthy subjects, each having three identical trials. Each trial consisted of three conditions: standing, treadmill walking, and treadmill walking with a closed-loop BCI. During the BCI condition, subjects used their brain activity to control a virtual avatar on a screen to walk in real-time. Robust procedures were designed to record lower limb joint angles (bilateral hip, knee, and ankle) using goniometers synchronized with 60-channel scalp electroencephalography (EEG). Additionally, electrooculogram (EOG), EEG electrodes impedance, and digitized EEG channel locations were acquired to aid artifact removal and EEG dipole-source localization. This dataset is unique in that it is the first published MoBI dataset recorded during walking. It is useful in addressing several important open research questions, such as how EEG is coupled with gait cycle during closed-loop BCI, how BCI influences neural activity during walking, and how a BCI decoder may be optimized.

## Background & Summary

Human walking is a complex task that involves supraspinal structures in the nervous system^1^. Trauma to these systems can lead to gait impairments, as in the case with stroke, the leading cause of serious long-term disability^2^. Thus, it is scientifically and clinically relevant to monitor human cortical activity during walking. Scalp electroencephalography (EEG) is a portable and relatively easy-to-use means to accomplish this goal by non-invasively measuring electrical brain activity. Numerous studies have recorded EEG from subjects performing seated tasks: reaching and grasping^3,4^, P300-related oddball detection^5^, and more recently walking^6–8^. However, EEG during walking has been given less attention partly because of logistical difficulties in practice and the potential for contamination from motion artifacts. Nonetheless, light-weight wireless EEG recording devices and better noise-removal algorithms have enabled some promising studies. EEG recording during walking has demonstrated features that differ from those recorded during standing, and more importantly, couple with the gait cycle^6^. There is plentiful evidence suggesting that EEG contains useful information about human walking^7–13^.

Here we present a MoBI dataset from eight healthy participants during a multi-trial treadmill walking protocol with and without BCI control of a virtual walking avatar. Data from 60-channel active EEG and goniometer measurements from 6 joint angles (bilateral hip, knee, and ankle) were recorded. Walking-related EEG artifact removal methods have been proposed in previous literature^10,14,15^. With careful setup, it has been shown that motion artifacts are negligible at lower speeds^16^. In this study, the treadmill speed was fixed at 1 mph (0.45 m/s) for all subjects during the walking period to minimize contamination of EEG by motion artifacts. Another reason for the slow speed is that future applications in gait rehabilitation are likely to involve slow speeds at least at the onset of the therapy. Additionally, four channels of electrooculogram (EOG), impedance values for the EEG electrodes at the start and end of experiments, and digitized EEG channel locations were also acquired to aid artifact identification and removal and/or EEG dipole-source localization. Impedance values were used to identify EEG channels with potential low signal quality. The digitization of electrode location had the dual purpose of aiding source analysis and ensuring repeatability of sensor cap placement from session to session. The EEG, EOG, and joint angle data were synchronized for subsequent segmentation into gait cycles.

Each subject participated in three trials of the same protocol. In each trial, the subject walked on a treadmill while watching a virtual avatar on a screen placed directly in front of the treadmill. In the *walk* phase, the avatar identically mirrored the lower limb movement of the subject by following the joint angles measured by goniometers. Then, in the *walk+BCI* phase, the hip, knee, and ankle joints of the avatar’s right leg were switched to be driven by the subject’s brain activity. A closed-loop BCI of the avatar was therefore created for this phase. Subjects were instructed to walk normally and consistently in the *walk* phase. In the *walk+BCI* phase, they were instructed to continue walking normally while trying to control their avatars. The BCI decoder was mathematically based on the unscented Kalman filter^17,18^; more information on this closed-loop BCI, including decoder calibration, is available in previous publications^19–21^.

Each trial consists of 20 min of walking and 4 min of standing (72 total minutes of EEG data for each subject). We have previously reported findings from this dataset, demonstrating significant difference in EEG when the participants engaged in a closed-loop BCI task^11^. To the best of our knowledge, the present data descriptor is the first publicly available EEG dataset involving walking, a task that needs careful preparation to ensure safety and data quality.

The richness and multi-trial feature of this dataset allow for further analyses of EEG and EEG-based BCI in order to:

- design offline decoders to continuously predict joint angles from EEG
- quantify the motion artifact in EEG during treadmill walking
- Investigate spectral features within the EEG that may be coupled with gait cycles
- measure the changes in BCI performance and/or EEG features across trials/days
- localize the EEG dipole sources during walking

## Methods

### Participants

Eight healthy individuals (three males, five females; aged from 19 to 29) with no history of neurological disease or lower limb pathology consented to participate in this study. All experimental protocols and informed consent (signed by all participants) were approved by the Institutional Review Board (IRB) at the University of Houston. All experiments were performed in accordance with the 45 Code of Federal Regulations (CFR) part 46 (“The Common Rule”), specifically addressing the protection of human study subjects as promulgated by the U.S. Department of Health and Human Services (DHHS).

### Experimental Protocol

Each participant visited our laboratory twice for three identical trials of data collection: two trials on the first day and one trial on the second day. Each subject had his/her two sessions scheduled within seven days.

**Figure 1:**
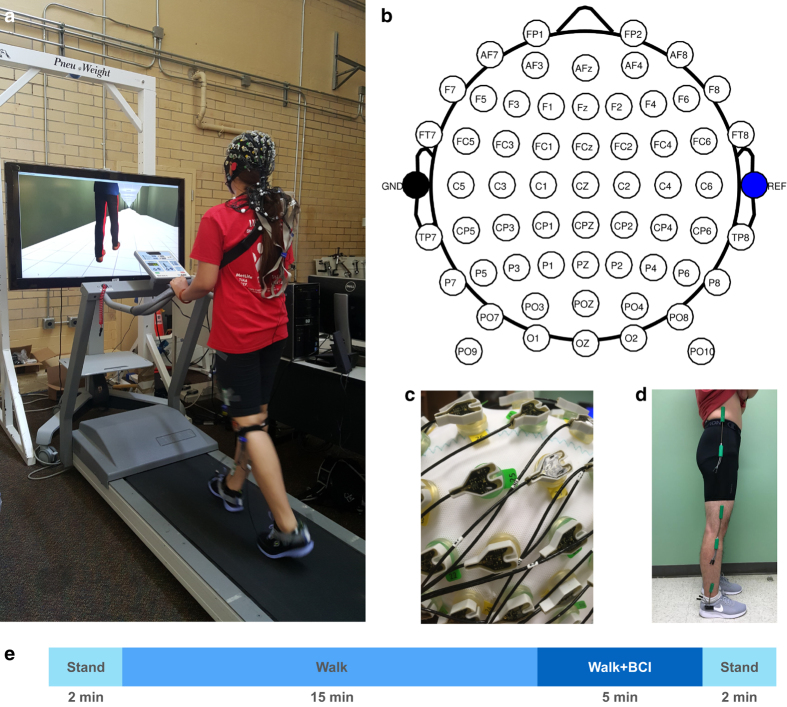
Experimental setup. (**a**) A subject walking on the treadmill, wearing EEG, EOG, and goniometer sensors. A 52-inch TV screen was placed in front to show a virtual avatar walking through a hallway. (**b**) Montage of 60 EEG channels. (**c**) Active EEG/EOG electrodes. (**d**) A goniometer unit, consisting of two endblocks connected by a spring. (**e**) Protocol timeline.

In each trial, subjects walked on a treadmill while controlling a virtual avatar on a computer screen. For safety purposes, all subjects were instructed to hold on to a safety handle directly in front of them while walking on the treadmill. The avatar was displayed at eye-level on a 52-inch monitor placed in front of the treadmill (Fig. 1a). The view in the virtual environment was set in a way that only the lower body of the avatar could be seen. The avatar has six degrees of freedom: sagittal flexion/extension in the hip, knee, and angle joints of both legs. As the subject walked on the treadmill, the avatar also advances through an infinitely long virtual hallway at roughly the same speed of the treadmill.

Subjects stood still for two minutes at the beginning and end of each trial for baseline EEG measurements (Fig. 1e). Between these two brief baselines, the subjects walked on a treadmill at one mile per hour (about 0.45 m/s) for two phases: *walk* and *walk+BCI*. In the 15 min of *walk* phase, the avatar accurately mirrored the lower limb movement of the subject by following the joint angles measured by the goniometers. In the 5 min of *walk+BCI* phase, the avatar’s right leg was switched to be driven by the prediction from the BCI decoder, forming a closed-loop brain control. The avatar’s left leg remained mirroring the subject’s left leg to provide a visual ground truth of the gait phase, and the BCI performance was visualized in the avatar’s right leg. Since the avatar’s left leg was always correct, it served as a helpful hint for the subject by providing a clear pattern for the brain-controlled right leg to follow.

### Data collection

Whole scalp 60-channel active EEG and 4-channel EOG activity were collected (ActiCap system, Brain Products GmbH, Germany) and labeled in accordance with the extended 10–20 international system (Fig. 1c). A RF wireless interface (MOVE system, Brain Products GmbH, Germany) was used to transmit data to the host PC at its default working band. The data was recorded at 100 Hz. The channel layout was modified from the standard Brain Products EEG cap setup as shown in Fig. 1b. Ground and reference channels were placed on the left and right earlobe (A1 and A2). Channels T7 and T8 were subsequently moved to FCz and AFz, where the ground and reference channels were originally positioned. This change allowed better sensor coverage of the parietal and frontal areas of the cortex. Sensors at FT9, FT10, TP9, and TP10 were moved to locations around the eyes (lateral sides of the eyes by the temples, and superior and inferior to the left eye) to capture ocular activities (electrooculogram, EOG): TP9, above the left eye; TP10, below the left eye; FT9, left of the left eye; and FT10, right of the right eye. Conductive gel (SuperVisc, Brain Products GmbH, Germany) was applied between sensors and the scalp to maintain impedances below 60 kΩ, and further reduced to below 20 kΩ in most channels. Channel impedances were recorded before and after each day’s data collection. Additionally, a camera-based 3D scanning system (BrainVision Captrak, Brain Products GmbH, Germany) was used to digitize the spatial positions of the EEG electrodes. If the scanned locations differed from the positions recorded in the previous session, the cap would be re-adjusted to maintain the same location across sessions and subjects. Sensor locations were re-scanned after adjustment in those cases.

**Figure 2:**
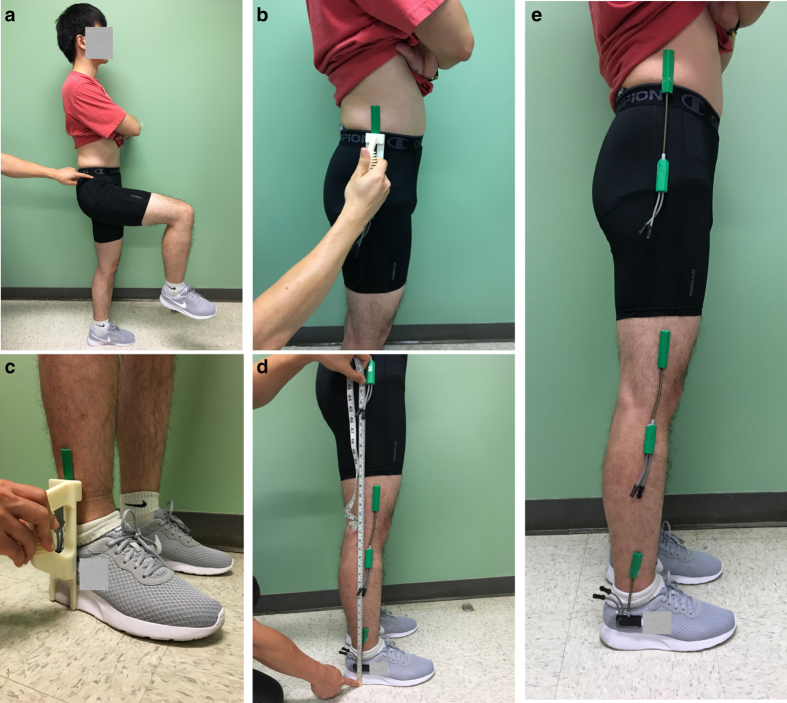
Setting up goniometers consistently across sessions and subjects. (**a**–**d**) show step-by-step demonstration of goniometer sensor setup. (**e**) Lateral view of the final configuration on one leg.

Bilateral joint angles on the legs (hip, knee, and ankle) in the sagittal plane were recorded by six goniometers (SG150 & SG110/A Gonio electrodes, Biometrics Ltd, UK) at 100 Hz (Fig. 1d). Subjects wore compression shorts for easier placement of the goniometers on the hips to reduce interference from clothing. We first attached all six goniometer sensors to customized 3D printed applicators (Fig. 2b and c), then donned them to the subject with double-sided medical tape. The custom applicators can maintain a neutral length in the goniometer springs and ensure repeatability. The 3D printed applicators are available by contacting the authors. The key procedures during goniometer setup are illustrated in Fig. 2. In each subject’s first session, we carefully identified the target position of each sensor by palpation (Fig. 2a). Specifically, the endblocks of the hip goniometers were placed on the lateral side of the pelvis (upper endblock) and femur (lower endblock) to monitor hip extension/flexion (Fig. 2b). Knee sensors were placed on the medial side of the femur (upper endblock) and tibia (lower endblock) for knee extension/flexion. Ankle sensors were also medially placed to the lower end of the tibia and medial part of the calcaneus to capture ankle joint plantar/dorsiflexion. The height of the ankle goniometers was about the same across subjects, therefore we designed the ankle applicator to position the ankle goniometers at the same height relative to the ground (Fig. 2c). Next, the height of the hip and knee goniometers was measured to be used as templates for the next session (Fig. 2d). Figure 2e illustrates the final configuration for one side of the body.

The goniometers were calibrated before the start of data collection in each session as follows:

1. zeroing: the goniometer measurements of the hip, knee, and ankle joints were reset to 0° while the subject was standing tall and upright.
2. obtaining the maxima: the goniometer measurements were recorded while the subject sat still in a chair (90° in the hips and knees), resting their feet on an inclined platform (43°), and later used as maxima angles. This platform was used for ankle joints because human ankles physically cannot reach 90° dorsiflexion.
3. linear mapping: the full range of motion for each joint was linearly interpolated between the measured zero and maximum angle endpoints: if a joint’s maximum angle was measured as *x* degrees during Step (2), a measurement of *y* degrees from this sensor would be linearly mapped to

$$y_{map}=\frac{y}{x}\cdot x_{0}$$

where *x*_0_=90° for the hip and knee joints, and *x*_0_=43° for the ankle.

### Eye artifact removal

The 4-channel EOG (Channel FT9, FT10, TP9, and TP10) were used as input to a real-time *H*^*∞*^ filter to remove ocular artifacts. It is based on an adaptive noise cancelling framework that updates at every sample^22^. During the closed-loop BCI, reference signals (EOG) were used to remove noise in the target signal (EEG). The reference signal *r* at time *t* is defined as

$$r_{t}={\left[\mathrm{TP}9_{t}-\mathrm{TP}10_{t},T9_{t}-\mathrm{FT}10_{t}\right]}^{T}$$

which is a two-element vector that describes the vertical and horizontal eye movements. A weight vector *w*_*t*_ was used to estimate the actual EEG signal *y*_*t*_ from measured EEG signal *s*_*t*_.

$$y_{t}=s_{t}-r_{t}w_{t}$$

The noise covariance matrix *P*_*t*_ was updated as

$${\tilde{P}}_{t+1}=\left[{{\tilde{P}}_{t}}^{-1}+\left(1-\gamma^{-2}\right)r_{t}{r_{t}}^{T}\right]+qI$$

$${P_{t}}^{-1}={{\tilde{P}}_{t}}^{-1}-\gamma^{-2}r_{t}{r_{t}}^{T}$$

where *γ* and *q* are manually chosen hyperparameters. *I* is the identity matrix. In this application, *q*=l*e*−10 and *γ*=5 were used. Finally, the weight vector *w*_*t*_ was updated:

$$w_{t+1}=w_{t}+\frac{P_{t}r_{t}}{1+{r_{t}}^{T}P_{t}r_{t}}y_{t}$$

This method was implemented for each EEG channel independently.

### Closed-loop BCI

In the 5-minute *walk+BCI* phase (see Fig. 1e), a closed-loop BCI was used to control the avatar. Fluctuations in the amplitude of slow cortical potentials in the delta band (0.1 – 3Hz) were used as the neural signal. An unscented Kalman filter (UKF) was implemented as a decoder that uses observation (EEG) to estimate the state variable (joint angle). UKF models the non-linear relationship between neural activities and joint angles. The state variable at time *t* was defined as

$$x_{t}={\left[\theta_{rh},\;\theta_{rk},\;\theta_{ra},\;\theta_{lh},\;\theta_{lk},\;\theta_{la}\right]}^{T}$$

where *θ* represents the joint angle at time *t*. Subscript *l* and *r* represents left and right; *h*, *k*, and *a* represents hip, knee, and ankle, respectively.

Normally, a Kalman filter^17^ starts with estimating the current state:

$$x_{t^{'}}=Fx_{t-1},$$

$$P_{t^{'}}=FP_{t-1}F^{T}+Q$$

where $x_{t^{'}}$ and $P_{t^{'}}$ are the predicted state and its covariance; *x*_*t*−1_ and *P*_*t*−1_ are the previous state and its covariance. Matrix *F* implements a linear movement model. *Q* is the covariance matrix of the noise.

In the update step, *H* is a matrix that implements a linear neural model that predicts the current EEG signal *z*_*t*_ from $x_{t^{'}}$:

$$z_{t}=Hx_{t^{'}}$$

The difference between the predicted EEG signal *z*_*t*_ and observed EEG *y*_*t*_ is used to correct the predicted $x_{t^{'}}$ to better correspond to the observation. This correction first requires calculating a matrix *S*, the covariance that describes the uncertainty in the predicted EEG:

$$S_{t}=HP_{t^{'}}H^{T}+R$$

where *R* is a noise term.

The amount of correction depends on the uncertainty of the current state estimate and the uncertainty in the prediction. A Kalman gain *K*_*t*_ is used to describe this weighted decision between $x_{t^{'}}$ and *z*_*t*_, and calculate the final estimation *x*_*t*_:

$$K_{t}=P_{t^{'}}H^{T}S_{t}^{-1}$$

$$x_{t}=x_{t^{'}}+K_{t}(y_{t}-z_{t})$$

Lastly, *P*_*t*_ is updated by $P_{t}=(I-K_{t}H)P_{t^{'}}$, where *I* is the identity matrix. Matrix *F*, *H*, *Q*, and *R* in the above equations were trained before the start of this real-time process.

UKF improves upon the classic Kalman filter by using a group of seeded samples (sigma points) to model the above steps nonlinearly. For a full description, please refer to this method’s original publication^17^ and our previous application^19^.

Although the BCI control only lasted 5 min, the UKF parameters (*F*, *H*, *Q*, and *R*) were trained during the previous *walk* phase using a closed-loop decoder adaptation (CLDA)^23^ algorithm. Under this training paradigm, the EEG and joint angle data were streamed to a buffer. Each minute, the buffered data were used to train a new set of *F*, *H*, *Q*, and *R*. Once a new set was trained, these matrices were updated by taking a weighted sum of the new values and the values from the previous update.

CLDA was activated in the *walk* phase and deactivated in the *walk+BCI* phase. Consequently, the UKF decoder was updated every minute during the *walk* phase, and then kept fixed during the *walk+BCI* phase. This practice separates the training data from testing and avoids overfitting.

### Code availability

The code we developed to record EEG/EOG and goniometer data, run the realtime BCI in the backend, and render the avatar on screen are integrated in a large C++ project. Please contact the authors directly for more technical information on the software.

## Data Records

All data files (archived in zip format) are available from *FigShare* (Data Citation 1). The data are stored in individual folders for each of the eight subjects (SL01-SL08) and each of their three trials (T01-T03). The folders are labeled as *SLxx-Tyy*, where *xx* is the subject number and *yy* is the trial number. Within each of these folders contains seven files:

### eeg.txt

The header for this file is one line indicating the number of channels recorded: “64 channels” (60 EEG and 4 EOG channels). The remaining rows contain timestamped EEG data tabulated in 65 columns: first column for the time stamp in seconds, columns 2–65 for the data from the 64 channels (channel labels are indexed in the file *impedances.txt)*. Note that the results in intermediate steps in the BCI decoder are not included. The final result of the decoder (predicted joint angles) is included in the *joints.txt*.

### joints.txt

This file contains the recorded and predicted joint angles. The header for this file consists of the first two lines. The first line states the number of joint angles that were measured (always 6 joints), followed by the 12 labels for the data table in the remaining lines below. The three-letter label *XYZ* corresponds to *X* being either G for goniometer-measured joint angle or P for predicted joint angle; *Y* being among H, K, or A for hip, knee, or ankle; and *Z* being either R or L for right or left. This line is the same across all subjects and trials.

The second header line indicates the values of the “joint factors”, the scaling factor obtained during goniometer calibration. They are denoted as *x* in Eq. 1.

The first column of the remaining lines is the time stamp (in seconds) and the remaining 12 columns correspond to the measured or decoded joint angle given by the labels. For example, the first column next to the timestamp column has label GHR, which indicates that it is the goniometer measurement of the right hip joint.

### conductor.txt

This file stores the timing of events during the data collection. The header for this file consists of the first two lines: the first line has the title plus the labels for the data columns (time, event); the second line states the number of times the CLDA decoder parameters were updated^19^. The data are in the remaining columns. The event IDs are included in Table 1.

### digitizer.bvct

*digitizer.bvct* is in XML format, and contains the digitized EEG electrodes 3D location data collected by the BrainVision Captrak software. The file includes the EEG cap size (circumference of the head in cm), the subject’s head shape (round or oval), and the Cartesian/polar coordinates for each electrode (*x, y, z, θ, φ, r)*. It is the same in Trial 1 and 2 for each subject because these two trials took place in one day. The EEG cap was kept in place throughout the two trials.

### impedances-before.txt & impedances-after.txt

The two files *impedances-before.txt* and *impedances-after.txt* contains the impedance of each EEG electrode measured with the ActiCap Control Box. The files include the indices, labels, and impedances for the EEG electrodes. As mentioned earlier, electrodes TP9, TP10, FT9, and FT10 were moved and instead used as the upper vertical EOG, lower vertical EOG, left horizontal EOG, and right horizontal EOG respectively. Same as *digitizer.bvct*, impedance files are the same in Trial 1 and 2 for each subject because these two trials took place in one day. *impedances-before.txt* was recorded before Trial 1 started, and *impedances-after.txt* recorded after Trial 2 ended.

## Technical Validation

### EEG data quality

We employed good measurement practices to ensure high quality data. Plastic twist ties were used to secure the EEG electrode cables to minimize their motion during walking. Electrode locations were scanned with a digitizer before each session to ensure alignment with the original template position (measured at the first session). After gelling, the correctness of the EEG data stream was validated by asking subjects to blink (observing for strong spikes in the frontal EEG channels) and to close their eyes (observing for alpha oscillations in occipital areas).

**Figure 3:**
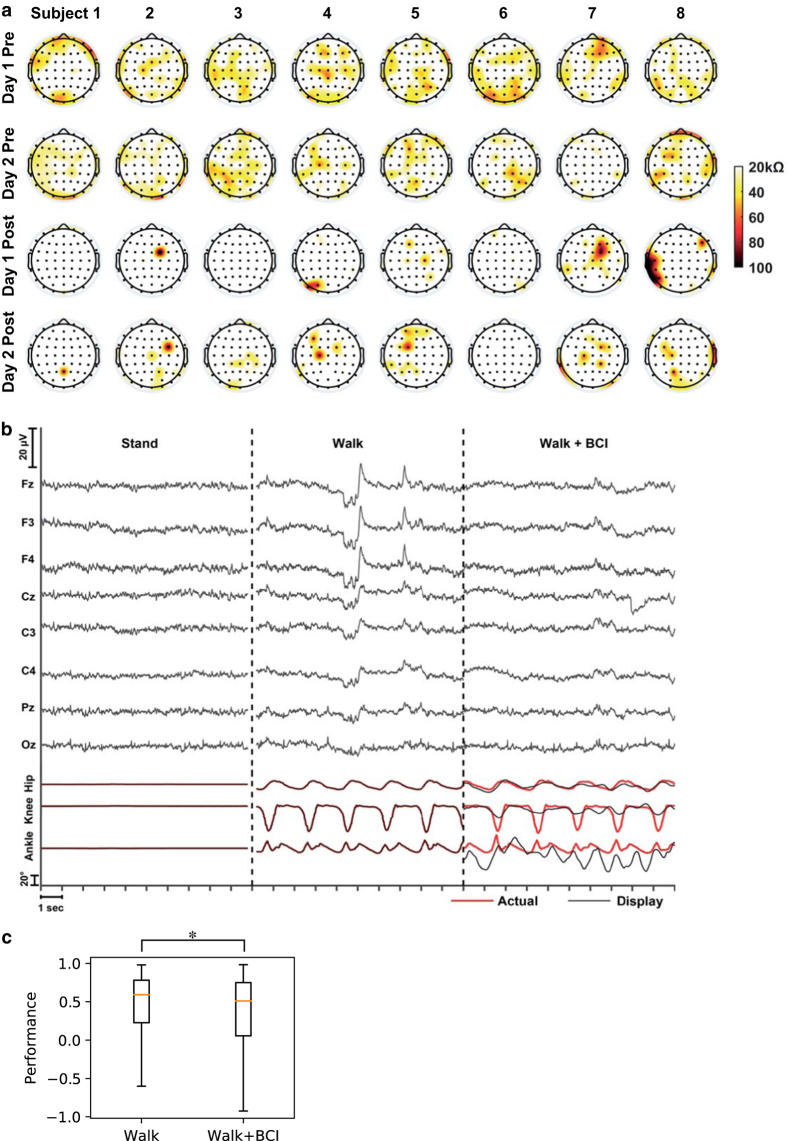
Validation of the quality of EEG, goniometer, and BCI. (**a**) Impedance of EEG electrodes before and after the experiments. Each subject was measured for four times as shown in the four rows: before (Pre) and after (Post) the experiment on both days. Scalp map outside the head circle was not shown to avoid incorrect representation due to interpolation at the area near boundary. (**b**) Sample raster plot of three 10-second periods during the *stand*, *walk*, and *walk+BCI* phases (from folder SL04-T03). Note that before the *walk+BCI* phase, the avatar-displayed joint angles (black line) always matched the actual measurement (red line). (**c**) Decoder performance measured by the r-values between the measured and predicted joint angles in the right hip.

The impedances for most channels were maintained below 60 kΩ in most channels. This was validated by measuring the impedances before and after the data collection. Figure 3a shows the color-coded impedance results: white indicates the impedance was 20 kΩ or below, yellow indicates around 40 kΩ, red indicates around 60 kΩ, and deep red and black indicates the impedance was above 60 kΩ. The impedances in all channels were set below 60 kΩ before the start of the experiment (empirically averaged to 23.7±13.4 kΩ across all sensors, subjects, and trials), but settled to below 20 kΩ in most channels by the end (averaged to 13.4±14.1 kΩ). Impedances in EEG sensors with gel usually decrease over time. A small number of channels had high impedance by the end of the experiment. Using a threshold of 60 kΩ at the end of the experiment, a total of 54 channels were identified with high impedance (using the *impedances-after.txt* files), which accounts for 5.1% of all the channels.

### Goniometer data quality

The goniometers were donned onto subjects with specially designed 3D-printed tools and procedures. To improve the consistency of sensor placement across days and subjects, we used the same sensor for each joint and recorded the distance (from the ground) of each goniometer after the sensors were attached. The goniometers were calibrated prior to data collection for each session to maintain the same absolute maximum range of motion across sessions and subjects. Figure 3b demonstrates three example periods of EEG and joint angle streams. The bottom three traces of the raster plot in Fig. 3b verify correct alignment of the hip, knee, and ankle goniometers.

### Data synchronization

Two sets of data were streamed during data collection: EEG/EOG and joint angles. The data collection software guaranteed that these two data streams were synchronized as they were recorded in pairs. In the *eeg.txt* and *joint.txt*, all samples are timestamped.

When the BCI was used to control the avatar (*walk+BCI* phase), the additional stream of predicted joint angles was also recorded. The predicted/displayed joint angle only deviates from the actual angle during this phase.

### BCI performance

The BCI performance can be evaluated by how closely the predicted joint angles resemble the measured joint angles. Mathematically, it was measured by their Pearson’s correlation coefficient (r-value), a scalar between −1 and 1. An r-value was calculated in each gait cycle. Figure 3c shows the right hip r-value distribution across all subjects and all trials. The *walk+BCI* box contains the r-values in all steps during that 5-minute phase, and the *walk* box contains the r-values in the steps in the last 5 min of the *walk* phase.

The result is reasonably good (median r-value equals 0.64 in the *walk* phase and 0.53 in *walk+BCI* phase) and in line with previous literature^19^. The performance during the *walk+BCI* phase was statistically lower than the *walk* phase (*P*<0.01). This is expected from the study design because the CLDA process was deactivated in the *walk+BCI* phase, which means the decoder stopped updating. It is worth mentioning that prolonged low performance in this phase may discourage the subjects and affect their levels of focus. The behavior of subjects remains to be quantified.

## Usage Notes

Most of the data in this MoBI dataset are readily available in tabular format stored in text files, which can be loaded easily with any common analytics software. We recommend EEGLAB (https://sccn.ucsd.edu/eeglab/index.php), an open source Matlab (The MathWorks, Natick, MA) toolbox, for EEG data processing. In addition to basic signal processing methods such as rejecting channels and frequency band filters, EEGLAB also comes with advanced EEG processing sub-toolboxes, such as Artifact Subspace Reconstruction (clean_rawdata(), https://sccn.ucsd.edu/wiki/EEGLAB_Extensions) for artifact removal and dipole fitting (DIPFIT, https://sccn.ucsd.edu/wiki/A08:_DIPFIT) for source localization. MNE (https://martinos.org/mne/stable/index.html) is an alternative free package developed in the Python environment.

Some of the common EEG processing steps include: noisy channel/epoch removal, common average referencing (CAR), independent component analysis (ICA), dipole fitting, independent component clustering, and time-frequency analysis (spectrogram). In walking-related studies, it is also common to segment the EEG data by gait cycles and then to average across the cycles. Gait segmentation can be accomplished by finding peaks in corresponding joint angle data which are synchronized with the EEG. Previous work usually incorporated some or all of these techniques in their EEG processing pipeline. For reference, see signal processing flowcharts such as Fig. 2 in Luu et al. 2017 (ref. 11), Fig. 2 in Gwin et al. 2010 (ref. 10), and Fig. 2 in Bulea et al. 2015 (ref. 13). Specifically, Luu et al. 2017 (ref. 11) may be helpful in understanding the typical usage as it used the same dataset.

## Additional information

**How to cite this article:** He, Y. *et al.* A mobile brain-body imaging dataset recorded during treadmill walking with a brain-computer interface. *Sci. Data* 5:180074 doi: 10.1038/sdata.2018.74 (2018).

**Publisher’s note:** Springer Nature remains neutral with regard to jurisdictional claims in published maps and institutional affiliations.

## Supplementary Material



## Figures and Tables

**Table 1:** Event ID legend for *conductor.txt*.

**Event ID**	**Meaning**
8	Start/stop training of the decoder
9	Quit program
10	Start/stop treadmill
11	Turn on/off the graphic display of decoding results during experiment (only visible to experimenters)
13	Decoder updated
16	Input signal (one EEG sample and one goniometer sample) registered
17	Turn on/off a graphic display that is obsolete in the present data (only visible to experimenters)
